# Weight/height^2^: Mathematical overview of the world's most widely used adiposity index

**DOI:** 10.1111/obr.13842

**Published:** 2024-10-10

**Authors:** Steven B. Heymsfield, John D. Sorkin, Diana M. Thomas, Shengping Yang, Moonseong Heo, Cassidy McCarthy, Jasmine Brown, Angelo Pietrobelli

**Affiliations:** ^1^ Pennington Biomedical Research Center, LSU System Baton Rouge Louisiana USA; ^2^ Baltimore VA Medical Center Geriatric Research, Education and Clinical Center Maryland USA; ^3^ Department of Medicine, Division of Gerontology, Geriatrics, and Palliative Care University of Maryland School of Medicine Maryland USA; ^4^ Department of Mathematical Sciences United States Military Academy West Point New York USA; ^5^ Department of Public Health Sciences Clemson University Clemson South Carolina USA; ^6^ Verona University Medical School Verona Italy

**Keywords:** adiposity, allometry, body composition, obesity

## Abstract

A footnote in Adolphe Quetelet's classic 1835 Treatise on Man described his algebraic analysis of how body weight ( W) varies with height ( H) in adult males and females. Using data on 12 short and 12 tall subjects of each sex, Quetelet established the rule that W is approximately proportional ( ∝) to *H* ^2^ in adults; that is, $W\propto H^{2}$ when $W\approx \alpha H^{2}$ for some constant α. Quetelet's Rule ( $W\propto H^{2}$), transformed and renamed in the twentieth century to body mass index ( $\mathrm{BMI}=W/H^{2}$), is now a globally applied phenotypic descriptor of adiposity at the individual and population level. The journey from footnote to ubiquitous adiposity measure traveled through hundreds of scientific reports and many more lay publications. The recent introduction of highly effective pharmacologic weight loss treatments has heightened scrutiny of BMI's origins and appropriateness as a gateway marker for diagnosing and monitoring people with obesity. This contemporary context prompted the current report that delves into the biological and mathematical paradigms that underlie the simple index $\mathrm{BMI}=W/H^{2}$. Students and practitioners can improve or gain new insights into their understanding of BMI's historical origins and quantitative underpinning from the provided overview, facilitating informed use of BMI and related indices in research and clinical settings.

**Abbreviations**
BMI: body mass index
H: height
NHANES: National Health and Nutrition Examination Survey
SE: standard error
W: weight

## 1 INTRODUCTION

The 1842 English translation of Quetelet's Treatise on Man contained an observation that still reverberates today: that in a cross section of adult males and females, body weight increases as height squared.^1^ Quetelet's Rule, now more than a century old and named “body mass index (BMI),”^2^ has now taken its place among other bodily vital signs as a global measure of health and disease risk.

The ascension of BMI to this distinguished rank as the most widely used shape index was a result of multiple reports showing it outperforms many competing indices^3^, ^4^, ^5^ and with ample criticism of underlying mathematical foundations^5^, ^6^, ^7^, ^8^, ^9^, ^10^ and medical contexts as marker of adiposity and health risks.^11^, ^12^, ^13^, ^14^, ^15^ These reports, some going back more than a century,^4^ are spread across many books and journals. There is no contemporary overview of underlying mathematical concepts nor the limitations associated with BMI. The aim of this report is to provide readers with the mathematical foundation, framed in a historical context, needed to critically apply and interpret BMI as a marker of adiposity and health risks in research and clinical settings. Additional suggested in‐depth readings are presented in Table 1.

**TABLE 1:** Selected references for further reading.

**Quetelet** Quetelet L, Knox R, Smibert T. A treatise on man and the development of his faculties, tr. (under the superintendence of R. Knox). [Ed. by T. Smibert]. People's ed. 1842. Eknoyan G. Adolphe Quetelet (1796–1874) The average man and indices of obesity. Nephrol Dial Transplant. 2008;23(1):47–51. doi:10.1093/ndt/gfm517.
**Allometry** Gayon J. History of the Concept of Allometry American Zoologist, Volume 40, Issue 5, October 2000, Pages 748–758, https://doi.org/10.1093/icb/40.5.748. Shingleton, A. (2010) Allometry: The Study of Biological Scaling. Nature Education Knowledge 3(10):2. Calder WA. Size, function, and life history. Mineola, N.Y.: Dover Publications 1996. Knut Schmidt‐Nielsen (Author) Scaling: Why is Animal Size so Important? 1st Edition.
**Weight–stature indices** Boyd E. Origins of the study of human growth; based on unfinished work left by Richard E. Scammon; edited by Bhim Sen Savara and John Frederick Schilke. Benn RT. Some mathematical properties of weight‐for‐height indices used as measures of adiposity. Br J Prev Soc Med. 1971;25(1):42–50. doi:10.1136/jech.25.1.42. Cole T. Weight‐Stature Indices to Measure Underweight, Overweight, and Obesity. In: Himes JE (ed.). In Anthropometric Assessment of Nutritional Status. Wiley‐Liss 1991; 83–111.
**Body mass index** Keys A, Fidanza F, Karvonen MJ, Kimura N, Taylor HL. Indices of relative weight and obesity. Journal of Chronic Diseases. 1972; 25: 329–43. Bray GA. Beyond BMI. Nutrients. 2023 May 10;15(10):2254. doi: 10.3390/nu15102254. PMID: 37242136; PMCID: PMC10223432. Flegal KM. How body size became a disease A history of the body mass index and its rise to clinical importance. Routledge Handbook of Critical Obesity Studies 1st Edition 2021.

## 2 EVOLUTION OF POWER LAW SHAPE INDEXES

### 2.1 Allometric foundation

Two measures of body size, weight and height, traveled a long road to arrive at their combined use in the form of BMI as a diagnostic tool for obesity and phenotypic marker of health risks.^2^, ^3^, ^4^, ^5^, ^11^, ^12^, ^16^ Quetelet's observations of body weight and height were reported in the larger context of his evolving views on social physics that included characterization of the *l'homme moyen* or average man.^17^ According to Quetelet, body weight ( W) in adults is approximately proportional ( ∝) to $H^{2}$ in adults. Specifically, $W\propto H^{2}$ when $W\approx \alpha H^{2}$ for some constant α. Quetelet's Rule is a specific example of the power rule relationship between a dependent variable, y, and independent variable X,

(1) $$y=\alpha X^{\beta }\varepsilon .$$

Log transforming both sides of Equation (1) yields

(2) $$\log_{10}y=\log_{10}\alpha +\beta \log_{10}X+\log_{10}\varepsilon .$$

The constant β is referred to as the scaling exponent or power, the constant α is referred to as the proportionality constant, and ε is the multiplicative error. The power law is widely used in biology as a tool to study the relations between an organism's size and their body shape, anatomy, composition, and metabolism. This area of biology is referred to as allometry,^18^, ^19^, ^20^, ^21^ a field founded by Otto Snell as a science in 1892.^22^ D'Arcy Thompson expanded Snell's work in 1917.^23^ The term allometry was coined by Huxley and Tessier in 1936 to describe their studies of the fiddler crab.^24^ Allometry is the study of the relationship between an organism's body size and the organism's shape, anatomy, physiology, and behavior. Three types of allometry are recognized, “ontogenetic” or “growth” that includes the study of trait relations in the same individual as they develop over time, “static” that evaluates traits in different individuals at the same stage of development, and “evolutionary” that describes trait relations across species.^19^, ^21^ Body mass index serves as an ontogenetic allometric measure when used to describe the relation between height and weight during growth beyond childhood and a static allometric measure when used to compare population characteristics.

**FIGURE 1:**
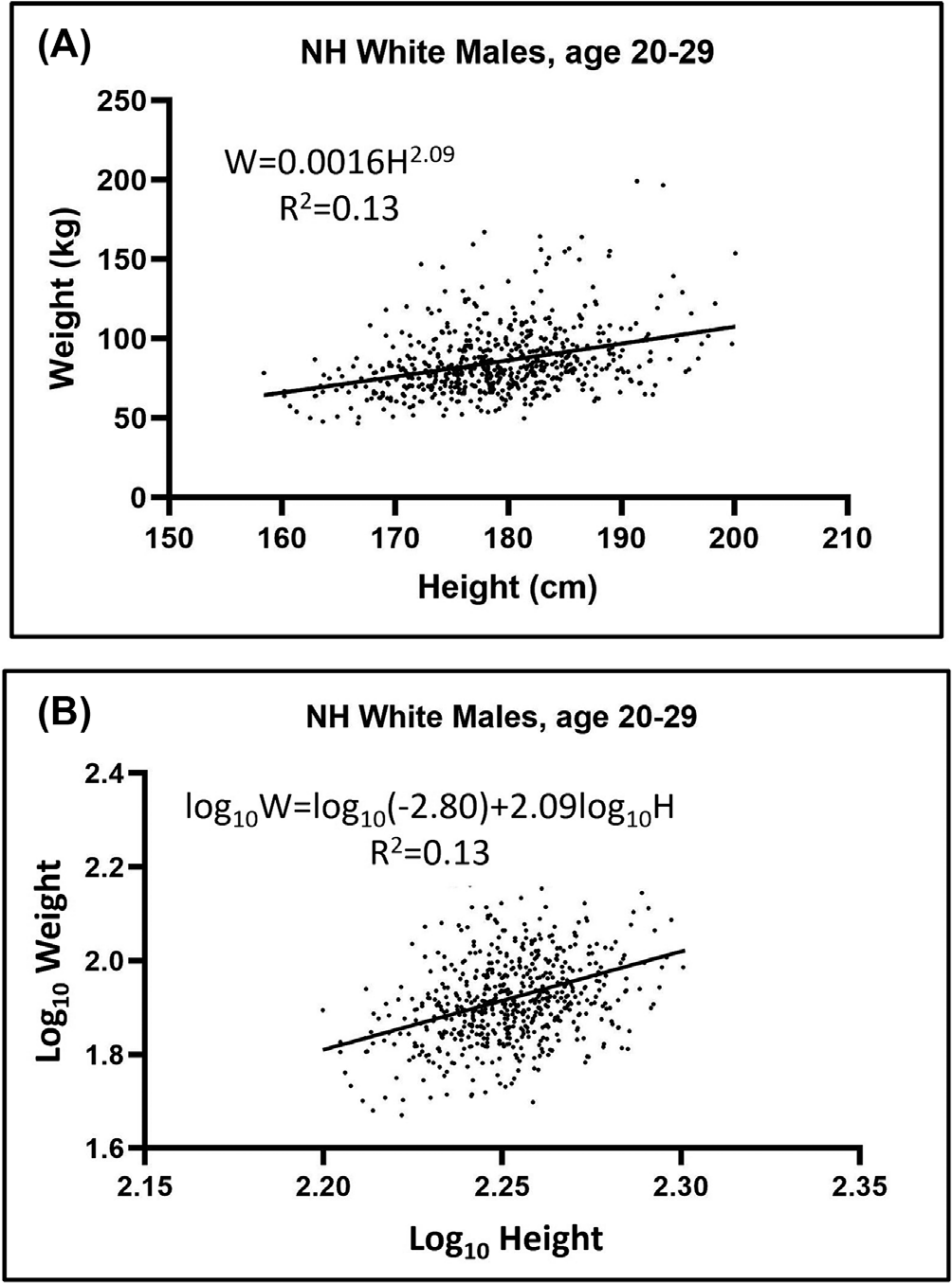
Body weight plotted against height in a sample of 614 young (ages 20–29 years) white male NHANES participants demonstrating application of (A) Equation (3) $(W=\alpha H^{\beta }\varepsilon$) and (B) Equation (4) ( $\log_{10}W=\log_{10}\alpha +\beta \log_{10}H+\log_{10}\varepsilon$). The developed regression equations with values for *β* are shown in each panel of the figure. The data in (A) were fitted with a power function and in (B) with a linear function as represented by the respective solid lines. Details of this sample can be found in reference.^25^

Weight as a function of height in young adult (ages 20–29 years) non‐Hispanic White male participants in the National Health and Nutrition Examination Survey (NHANES) is shown in Figure 1.^25^, ^26^ We can convert the general allometric models to a form relevant to the current context by setting y=W and X=H in Equations (1) and (2) where W represents weight in kilograms and H represents height in meters,

(3) $$W=\alpha H^{\beta }\varepsilon \;\text{and}$$

(4) $$\log_{10}W=\log_{10}\alpha +\beta \log_{10}H+\log_{10}\varepsilon$$

Panel A of the figure shows a plot of the general form depicted by Equation (3). Fitting Equation (4) using ordinary least square regression resulted in a height power, β, equal to 2.09 as shown in panel B of the figure. We thus find that in this sample of young adult males that weight scaled to height with a power of about 2 and thus $W\approx \alpha H^{2}$. Quetelet used an algebraic solution to derive his value of β in a footnote of his publication as shown in Table 2.^17^

**TABLE 2:** Quetelet's derivation of *W* ∝ *H* ^2^ in adults.^a^

Quetelet concluded in the 1842 English translation^1^ of his 1835 treatise Sur l'Homme^17^ that “*if we compare two individuals who are fully developed and well‐formed with each other, to ascertain the relations existing between the weight and stature, we shall find that the weight of developed persons, of different heights, is nearly as the square of stature*.” Quetelet's analysis was based on “*12 of the smallest individuals of both sexes, and 12 of the largest, submitted for observation*”:		
Men	Stature (m)	Ratio of weight to stature (kg/m)
The smallest	1.511	36.7
The largest	1.822	41.4
Women		
The smallest	1.456	35.6
The Largest	1.672	38.0

Adapted from Quetelet's Sur l'Homme English Translation, page 66.^1^

*Calling *t* and *T* the statures and *p* and *P* the corresponding weights of the smallest and the largest individuals, we have, in fact, almost exactly, *t*: *T*:: 5: 6, by the numbers of the first column belonging to men, and *p/t*: *P*/*T*:: 5: 6 for those of the second, from which we find that *t*: *T*:: *p/t*: *P*/*T*, or, in other words, *t* ^*2*^: *T* ^2^:: *p*: *P*. It is the same with the numbers belonging to females.

To expand on Quetelet's footnote, we see from the table that t=1.511m; T=1.822m and $\frac{p}{t}=36.7\frac{\mathrm{kg}}{m};\frac{P}{T}=41.4\frac{\mathrm{kg}}{m}$ Quetelet observed in men that $\frac{t}{T}\approx \frac{1.511\;m}{1.822\;m}\approx 0.83\approx \frac{5}{6}$ and $\frac{\left(\frac{p}{t}\right)}{\left(\frac{P}{T}\right)}\approx \frac{36.7\;\frac{\mathrm{kg}}{m}}{41.4\;\frac{\mathrm{kg}}{m}}\approx 0.89\approx \frac{5.3}{6}\approx \frac{5}{6}$ Therefore, $\frac{t}{T}\approx \frac{5}{6}$ and $\frac{\left(\frac{p}{t}\right)}{\left(\frac{P}{T}\right)}\approx \frac{5}{6}$ Then, $\frac{\left(\frac{p}{t}\right)}{\left(\frac{P}{T}\right)}\approx \frac{\mathrm{pT}}{\mathrm{Pt}}\approx \frac{5}{6}$ and $\frac{\mathrm{pT}}{\mathrm{Pt}}\approx \frac{5}{6}\approx \frac{t}{T.}$ It follows that $\frac{\mathrm{pT}}{\mathrm{Pt}}\approx \frac{t}{T}$ and thus $\frac{p}{P}\approx \frac{t^{2}}{T^{2}}$

Weight in Quetelet's short men thus related to weight in their taller counterparts in proportion to their respective heights squared. The same observation applied in women.

^a^ Modified from Flegal KM. How Body Size Became a Disease: A History of the Body Mass Index and Its Rise to Clinical Importance. In: Gard M, Powell D, Tenorio J (eds.). *Routledge Handbook of Critical Obesity Studies*. Routledge 2021; 23–39.

**FIGURE 2:**
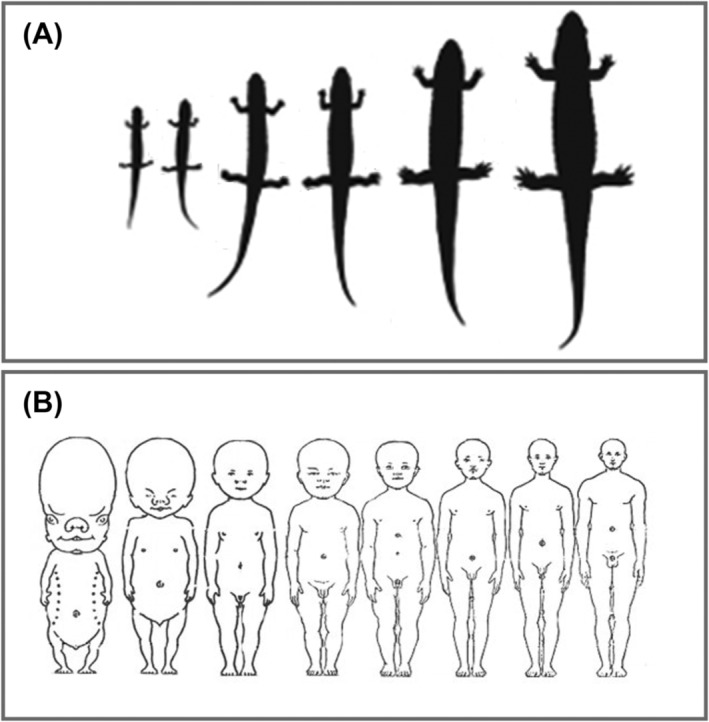
(A) Ontogenetic allometry in salamanders that display isometric growth with body proportions remaining stable and (B) humans that exhibit allometric growth during which body proportions change.^21^, ^27^

**FIGURE 3:**
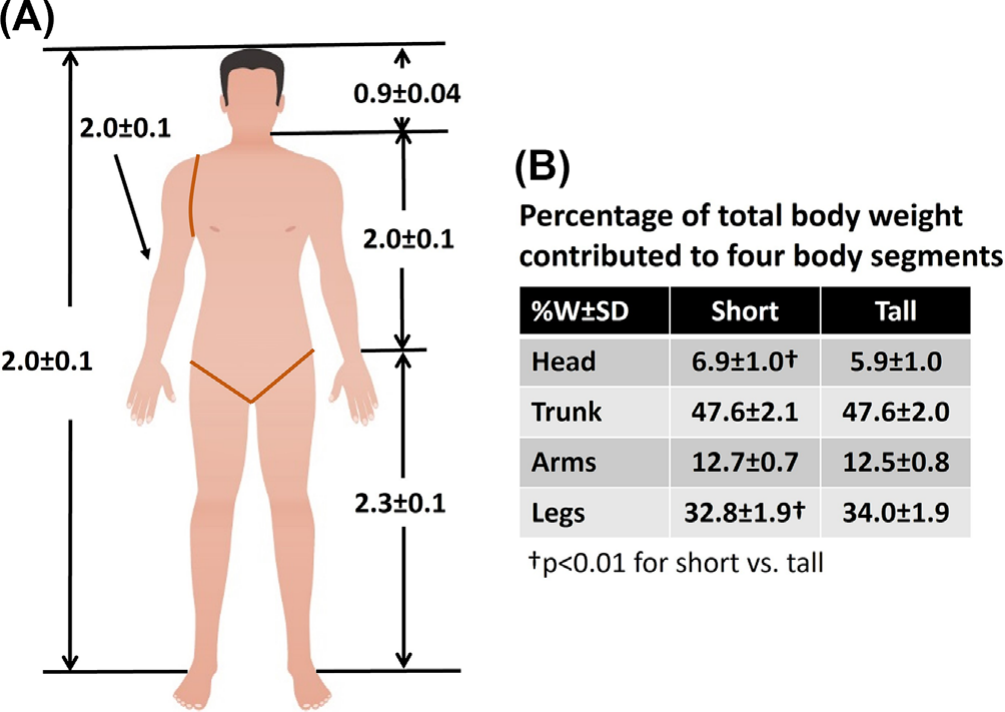
(A) A list of exponents (*β* ± SE) for different body segments that can be used to determine the relation between the weight of the segment *W* and the length of the segment *H* (i.e., weight of the segment ∝ length^*β*^). For example, the weight of the leg in adult males presented in Figure 1 was proportional to its length raised to the power of 2.3 (i.e., leg weight ∝ leg length^2.3^) where overall weight was proportional to the square of overall height (i.e., body weight ∝ overall height^2^).^25^, ^28^ Because the legs of tall people are generally a larger fraction of overall height than in short people, the percentages of body weight of regions such as the leg will therefore be greater in people who are tall versus those who are short (B); the opposite is true for head and neck mass (head–neck weight ∝ head–neck length^0.9^) as shown in the table. Tall adults are therefore not isometric or “geometric” replicas of short adults. See Supporting Information I for additional details.

The data presented in Figure 1 is an example of static allometry showing how weight scales to height in a cross‐sectional sample of males who are similar in race/ethnicity and age. When directly proportional relationships are preserved in a linear manner with greater body size the scaling is referred to as “isometric.” For example, consider a square of side length, l. The area of the square is $l^{2}.$ If we increase the side length by a factor of c, then the new area is going to be a direct proportion of the old area: $c^{2}l^{2}.$ Likewise, the new volume of the cube generated by the original square is going to be $c^{3}l^{3}$. In each case, the new area is directly proportional to the old area. In general, isometric scaling assumes that measurements of large bodies (length, area, volume) are simply directly proportional to the measurements of small bodies. As salamanders grow, the ratio of the lengths of their body parts is nearly constant. For example, in panel A of Figure 2, the length of the salamander's tail is always approximately one‐half the length of the entire salamander, regardless of the overall size of the animal. We refer to deviations from isometry as “allometric” scaling. In the case of deviation from isometric scaling, measurement of a larger body size will not be directly proportional to measurements of a smaller body size. Compared with salamanders, humans exhibit allometric growth with body proportions changing from birth through adolescence. For example, as shown in panel B of Figure 2, a baby's head is very large relative to overall height. As the baby grows towards an adult size, the baby's head decreases relative to overall height. Quetelet understood these allometric relationships, stating that “*If man increased equally in all his dimensions, his weight at different ages would be the cube of his height. Now, this is not what we really observe*,” anticipating the formal development of allometry by Otto Snell 57 years later.^22^ Then, moving on specifically to adults and static allometry, Quetelet stated that “*If we compare two individuals who are fully developed, and well‐formed with each other, to ascertain the relations existing between the weight and stature, we shall find that the weight of developed persons, of different heights, is nearly as the square of stature*.”^1^ As recognized by Quetelet, allometric scaling is present in adults as shown in panel A of Figure 3 for the White males presented earlier; the exponent, 2.3 relating leg length to leg weight (leg weight ∝ leg length^2.3^), is larger than the exponent relating overall height to overall weight (overall weight ∝ height^2.0^), torso length to torso weight (torso weight ∝ torso length^2.0^), arm length to arm weight (arm weight ∝ arm length^2.0^), or head–neck length to head–neck weight (head–neck weight ∝ head–neck length^0.9^). These regional scaling effects translate to different body proportions in tall and short adults: In tall adults, the legs are generally a larger proportion of total height then they are in short adults (panel B). As a result, people who are tall have a larger proportion of their weight in their legs, a similar proportion in their arms and trunk, and a smaller proportion in their head and neck than those who are short.^28^, ^29^ Adults who are tall are thus not isometric representations of people who are short.

Quetelet's observation that weight is proportional to height squared in adults was soon recognized by his contemporaries. The American astronomer Benjamin A. Gould, evaluating data from 16,377 Civil War soldiers in 1869,^30^ also reasoned that “*did the average proportions remain unchanged in men of different stature, we might expect their weights to be to one another as the third power of their heights*.” Confirming Quetelet's observation, Gould found that “*we are irresistibly led to the singular and interesting discovery that the mean weights*” “*appear to vary strictly as the squares of the statures*.” Gould's original data and derivation are presented in Supporting Information II.

Quetelet's observation that $W\approx \alpha H^{2}$ in adults is considered among the first allometric laws in biology.^31^ Allometric research today usually extends beyond these kinds of empirical relations to causal mechanisms. However, the physical and metabolic mechanisms driving the consistent adult human whole body and regional scaling patterns are largely unknown at present and offer an opportunity for future research.

### 2.2 Proliferation

Artists, anatomists, and students of growth in the late eighteenth century through the mid‐twentieth century increasingly became interested in studying human form and body build as quantified with power‐type indices that included values of β in the ratio $W/H^{\beta }$ ranging from whole integers of 1 to 3 and their associated fractions. Beginning with β=1, the weight–stature ratio ( W/H) was advocated by the anthropologist Richard Pearl in 1940 as a “suitable” measure of body build.^32^, ^33^ Charles R. Bardeen inverted the weight–stature ratio, advancing the “ H/W index of build” in 1920 as a means classifying the proportions of the human body.^34^ With β=2, Quetelet's “index” ( $W/H^{2}$), as it became known, was explored or critically examined by Davenport,^35^ Martin,^36^ Billewicz et al.,^37^ Khosla and Lowe,^38^ Florey,^39^ and Keys et al. who renamed Quetelet's index BMI in 1972.^2^ The French naturalist Buffon first suggested $\frac{W}{H^{3}}$ (i.e., β=3) in the late 1700s.^40^ The Italian anthropologist Rodolfo Livi developed L'indice ponderale, $100\times W^{\frac{1}{3}}/H$, in 1897 as a measure of body build and fat proportions.^41^ Variations of Livi's ponderal index soon followed with Rohrer (1908, $100\times W/H^{3}$, Corpulence or Rohrer Index), Pirquet^42^ (1922, Pelidisi ratio, $W^{\frac{1}{3}}/\mathrm{SH}$ where SH is sitting height),^32^ Pfaundler^43^ (1916, $H/W^{\frac{1}{3}}$),^4^ and later Sheldon^44^ (1940, again $H/W^{\frac{1}{3}}$). The proliferation of weight–stature indices, with only a partial listing and bibliography presented here, came with legions of advocates who presented formidable biological and mathematical arguments on behalf of their weight–stature models.

### 2.3 Benn index

An influential index suggested by Benn in 1971 specified a population‐specific value of $\beta \;\text{in}\;W/H^{\beta }$. Benn's focus at the cusp of the obesity “epidemic” was on the mathematical properties of relative weight and power‐type indices as measures of adiposity. Benn stated that a need exists for a “*reliable, practicable, and generally acceptable index of obesity*.”^3^ Body weight and height were easily acquired in epidemiological studies, and according to Benn, the disadvantages of developed indices would be offset by their “*great merit that the measurements themselves can be made easily, quickly, and with a fair degree of accuracy*.” Benn proposed that a “good” index of obesity should meet two main criteria: independence from height and high correlation with measures of adiposity. Benn favored a power‐type index in the form of $W/H^{\beta }$ with the value of β derived on the specific population of interest. Benn proposed an approach for deriving a value of β±SE, where SE is the standard error, that would be independent of height and maximally correlated with adiposity.^3^, ^5^ Benn's approach requires that adiposity in the evaluated sample be independent of height, a key assumption largely supported by earlier and more recent studies in adults.^3^, ^5^, ^37^ Later investigators referred to the family of population‐specific forms of $W/H^{\beta }$ as Benn's Index.^45^ Quetelet's Index is a special case of Benn's Index in which β=2. An unappreciated concern voiced by Benn and others up to the 1970s was the lack of highly accurate methods for estimating adiposity.^3^, ^39^ While early investigators could establish which power‐type indices were independent of height, determining associations with adiposity was more difficult and often evaluated with measures of total body fat such as skinfolds.^3^, ^5^, ^46^ Brozek and his associates advanced “hydrodensitometry,” initially developed by Behnke et al. in 1942,^47^ as a means of characterizing adiposity in the 1950s,^48^ although investigators such as Billewicz^37^ in 1962 considered “*estimates of total body fat based on densitometric measurements far from accurate*.” Body density, measured with Brozek's underwater weighing method,^48^ is inversely correlated with the proportion of body weight as fat. Benn's classical 1971 report described adiposity as the log‐transformed sum of three skinfold measurements, triceps, subscapular, and suprailiac. Problems associated with skinfold evaluations include the need for exacting training and execution of the measurements; results obtained from those who were untrained or not well trained were neither accurate nor dependable. Additionally, the equations used to develop the conversion from skinfolds to body fat were derived from hydrodensitometric measurements. The accuracy of the formulae for predicting total body fat mass in a given patient was thus only as good as the degree to which that person was represented by the pool of people who underwent the hydrodensitometric measurements. Critical analyses of weight–stature indices up to the 1970s thus often relied on methods that were not viewed as accurate or available means of phenotyping a person's or group's adiposity level. It was only later, beginning in the 1970s, that modern methods for evaluating adiposity such as computed tomography (1973), magnetic resonance imaging (1977), dual‐photon absorptiometry (1981), and bioimpedance analysis (mid‐1980s) became available to investigators.^49^

### 2.4 Body mass index

Keys et al. published their seminal study of relative weight indices of obesity in 1972.^2^ The aim of Keys' study was to find the optimum index that removed “*the dependency of weight on height*” and that maximally associated with “*relative obesity or body fatness*.” Keys et al. studied two Benn‐type indices, *W*/*H* and *W*/*H* ^2^, with β set as two whole integers ( β=1,2) and a third power‐type index, Livi's Ponderal Index ( $W^{\frac{1}{3}}/H$). In addition to the three power‐type indices, Key's et al. included in their evaluations a relative weight index expressed as a percentage of average weight for height and age.^2^ Acknowledging the past studies of Quetelet's Index, Keys et al. “*proposed that this ratio*, $W/H^{2}$, *be termed body mass index*.” Insurance companies started to develop actuarial tables at the beginning of the twentieth century that established a person's mortality risk based on their “relative weight.” The “standard height–weight” tables published in 1912 by the Joint Committee on the Medical‐Actuarial Mortality Investigation included average weights tabulated by sex, age, and height.^50^ Expressed as a percentage, a person's relative body weight could be classified as underweight or overweight. Later, The Metropolitan Life Insurance Company published “ideal” body weight tables, one for women in 1942 and one for men in 1943. This was followed by the publication of “desirable” body weight tables, one for men and one for women in 1959. A third set of tables was published in 1983 entitled Height–Weight tables without any attached adjective. In their day, these tables were arguably the de facto height for weight guidelines. A fourth set of tables, “desirable weight” tables, was published in 1999, but these tables were less influential than the prior sets. All four sets of tables gave a range of weights for a given height, according to body frame, small, medium, and large.^51^, ^52^, ^53^ The desirable weight tables published in 1959 by the Metropolitan Life Insurance Company^53^ were widely available at the time of Keys' research. The work of Keys, an examination of Metropolitan Life Insurance Company Tables, and other data, was used by Reubin Andres as he produced the Gerontology Research Center Table which gave weight ranges associated with less than average mortality in 1‐in height increments.^54^ Viewed from an allometric perspective, the Metropolitan Life Tables, and the Gerontology Research Center Table developed by Andres, are expressions of power functions of the form *W* = *α*(*H*)^*β*^. Using the 1959 Metropolitan Desirable Weight tables as an example, let *W* (given in pounds) be desirable weight and *H* height (given by inches of height from 4′10″ to 6′0″ for women and 5′2″ to 6′4″ for men). Weight and height were given assuming indoor clothing with shoes on. Viewed from an allometric perspective, these charts form the basis of desirable weight power functions of the form *W* = *α*(*H*)^*β*^; that is, *W* is desirable weight and *H* is height in the 1959 tables evaluated as measured in indoor clothing with shoes on.^54^ Solving the power law model for each sex and frame size yields powers of height ranging from 1.79 to 1.91 (Supporting Information III), not far from about 2 as observed by Quetelet.^1^ Body mass indices with the lowest mortality as indicated in the 1959 desirable weight tables as derived from these models range from a low of 19.4 kg/m^2^ for women with a small frame to 24.5 kg/m^2^ for men with a large frame, a span generally consistent with contemporary healthy BMI ranges. The desirable weight tables were heavily criticized for a number of reasons including lack of clothing standardization during measurement of height and weight, no information was provided on how frame size was established, or how frame size could be estimated in the clinical setting, limited associations between relative weight and adiposity, and design concerns related to the studies forming the basis of weight–height tables.^12^, ^55^, ^56^, ^57^ Keys et al. derived their own reference values for relative weight from updated 1912 standard height–weight tables as part of their epidemiological studies related to coronary heart disease in 1966.^58^

The study sample evaluated by Keys et al. comprised 12 groups of men (*n* = 7425) with diverse occupations and nationalities who ranged in age from 18 to 60 years and who had an average BMI of less than 27 kg/m^2^. Adiposity was quantified as the sum of two skinfolds (triceps and infrascapular) measured with calipers and body density measured with underwater weighing, the hydrodensitometry method introduced earlier by Behnke et al.,^47^ and the implementation of the technique pioneered by the authors^48^ and executed with great attention to detail in their laboratory. Body density was measured only on a subgroup of the men, 180 college students ages 18–24 years and 249 executives ages 49–59 years.

Meeting Benn^3^ and Key's criteria for a stature‐independent shape index, percentage of average weight, and $W/H^{2}$ had the lowest correlations with height across the multiple male samples. With respect to adiposity, Keys et al. found that Livi's Ponderal Index ( $\;W^{\frac{1}{3}}/H$)^41^ had the lowest correlations with the sum of skinfolds, although the authors noted that even the highest correlations for several indices (e.g., $W/H^{2},$ evaluated students, *r* = 0.85; executives, *r* = 0.66) left about one‐third to one‐half of the variance in adiposity unexplained. Body density was least correlated across the samples with Ponderal Index, although Keys et al. again noted that $W/H^{2}$ accounted for little more than half of the total variance present in regression analyses. The authors concluded that $W/H^{2}$, or Quetelet's Index renamed BMI, “*seems preferable*” over the other three indices as it performed better than the two power‐type indices ( W/H and $W^{\frac{1}{3}}/H$) and relative weight against measures of adiposity, that it is simple to calculate, and that unlike percentage of average weight, BMI is not population or adult‐age specific.

The study of Keys et al. published in 1972 was prescient: 1 year later, in 1973, a Fogarty International Center Conference held at the National Institutes of Health addressed the rising prevalence obesity as a “*significant public health problem in the United States and in most other affluent nations*.”^59^ The final conference report included a suggested reference guide for adult weight in relation to height based on the 1959 Metropolitan Life Insurance Company desirable weight tables. The reference guide could be used to establish an individual or group's deviation from an acceptable weight and provide uniformity in reporting and tracking overweight at the population level. Although Keys was present at the meeting, only one table in the conference report described selected BMIs from his 1972 publication^2^ and note was made of the low correlations present between W/H^2^ and adiposity; the conference authors concluded that any “*estimate of obesity from height and weight leaves a considerable amount of uncertainty estimating body fat*.”^59^ Accordingly, discussion centered on the distinction between “overweight” as defined by measured weight and height and “obesity,” an excess of body fat estimated with methods such as skinfolds and hydrodensitometry. Note, however, was made of the range of BMIs associated with lowest mortality based on sex and frame size as published in the 1959 desirable weight tables.^57^, ^59^

**FIGURE 4:**
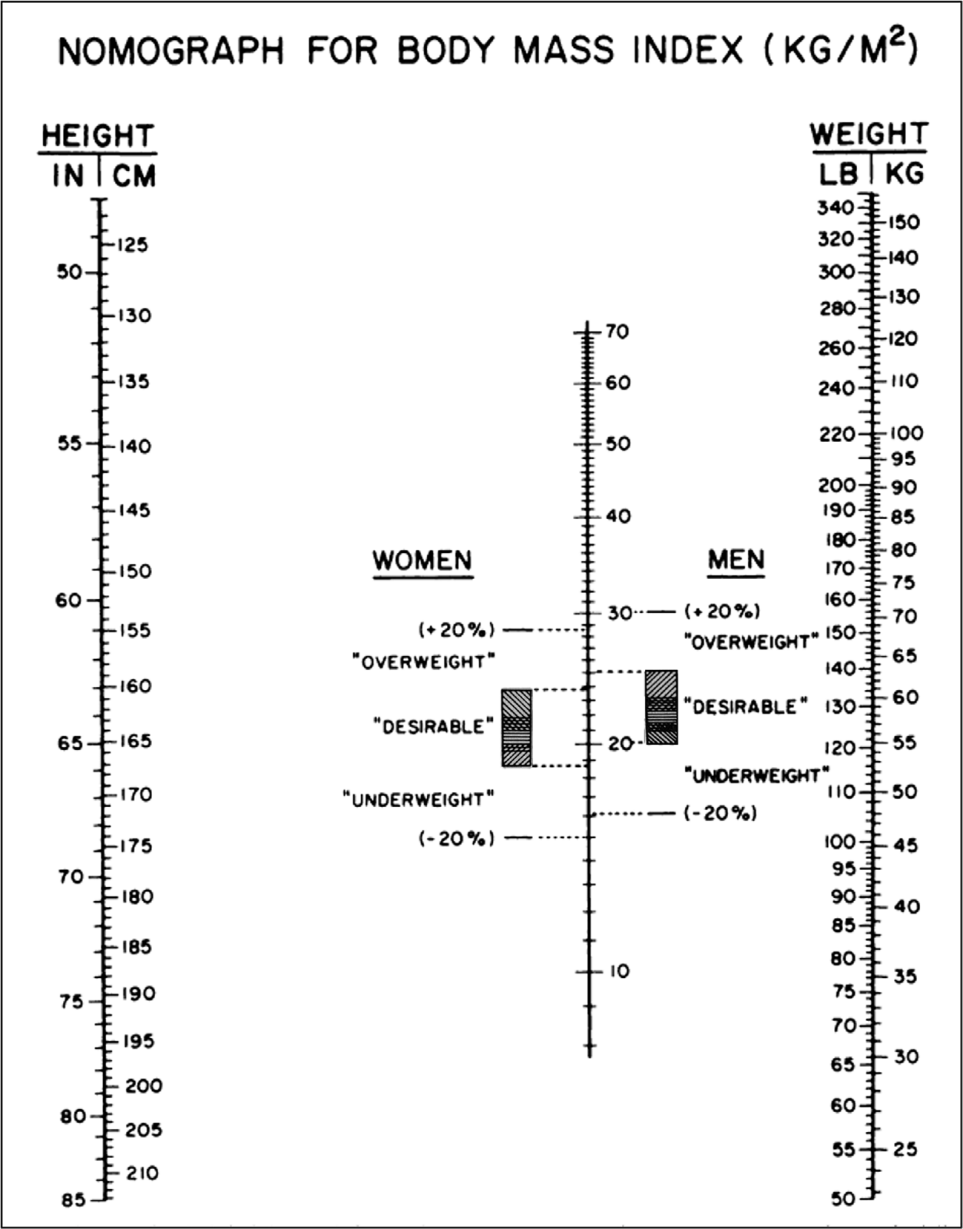
Nomograph for converting weight and height to BMI as reported by Thomas et al.^61^ The ranges suggested as “desirable” are based on life insurance data,^53^, ^64^ and the shading within the desirable ranges suggests adjustments for frame size: horizontal shading for “medium frame,” upper left to lower right diagonal shading for “large frame,” and lower left to upper right for “small frame.” Levels ± 20% were considered outside of the desirable range. This illustration was published in Thomas.^61^

Concerns with the rising prevalence of obesity in the USA accelerated in the mid‐nineteen seventies,^13^, ^60^ although BMI had not yet displaced other competing adiposity phenotyping methods. In 1976, Thomas et al. noted that BMI “*has received little use in clinical settings*” because “*division of weight by the square of height*” is mathematically “*demanding*.”^61^ Digital calculators were to become available to consumers towards the late nineteen seventies,^62^ making BMI less challenging to calculate by practitioners and the general public. Thomas and his colleagues proposed solving this problem by publishing a nomograph from which BMI could be easily ascertained from weight and height (Figure 4). Thomas et al., suggesting that the nomograph could be “photocopied” for clinical applications, included BMI ranges tethered to life insurance tables^53^, ^61^ with “desirable” weights spanning roughly BMIs of 19–25 kg/m^2^. Two years later, Bray published a refined “nomogram” with an “acceptable” BMI range linked to life insurance tables^53^, ^63^ of 20–25 kg/m^2^ for males and 19–24 kg/m^2^ for females, overweight as between the upper limits of normal BMI and a BMI of 30 kg/m^2^, and obesity as BMI > 30 kg/m^2^.^59^, ^64^ With BMI still not firmly in place, Bray included a table with weight‐for‐height guidelines from which a person's relative weight could be calculated in the proceedings of the 1973 Fogarty Conference on Obesity in Perspective.^59^ In 1981, Garrow suggested “convenient” *W*/*H* ^2^ cut points with four grades ranging from grade 0 (*W*/*H* ^2^, 20–24.9 kg/m^2^) to grade III (*W*/*H* ^2^ > 40 kg/m^2^).^65^ Garrow noted, however, that “*readers with mathematical inclinations may be worried*” that “*a weight divided by the square of a length seems to denote a pressure, that is difficult to relate logically to obesity*.” Garrow went on to suggest that *“it would be easier to accept W/H* ^*3*^ *which implies weight divided by a volume, which is a unit of density*” and thus a measure of adiposity. Garrow concludes his justification of classifying obesity as *W*/*H* ^2^ by noting Benn's analyses of weight–stature indices^3^ stating that “*Quetelet's index will probably be as good as any if one is forced to choose blindly*.” Current national and international healthy weight guidelines based in BMI evolved from these early discourses on the best index to apply in evaluating the growing prevalence of obesity and other weight‐related medical conditions.^11^, ^12^ Studies published in the five decades that followed associated BMI with not only adiposity, but many other outcome‐related endpoints in varied populations. The study of Keys et al.^2^ that laid the foundation for these efforts has now been cited over 3000 times in journal articles since publication in 1972.

## 3 DERIVING AN OPTIMUM WEIGHT–STATURE INDEX

Benn^3^ and Keys et al.^2^ searched for optimum powers of height that when incorporated into a power‐type index ( $W/H^{\beta })$ met two criteria, independence from height and maximum correlation with adiposity. How can values for β be derived in a study sample that meet these two criteria? Is 2.0 the optimum value for β as reported by Keys and his colleagues?^2^ Cole summarized three approaches for developing values for β in a comprehensive 1991 review of weight–stature indices.^5^ In this section, we describe Cole's three suggested approaches for developing values for *β*.

**FIGURE 5:**
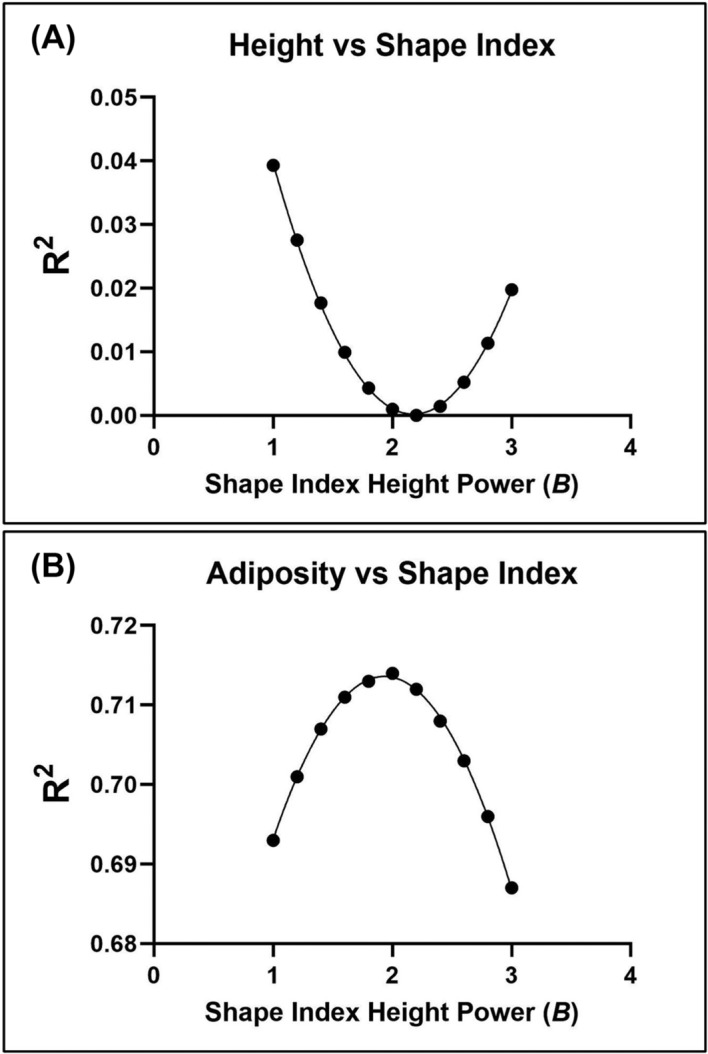
(A) Correlations (*R* ^2^) between height and increments in shape indexes in the form of *W/H* ^*β*^ and (B) correlations (*R* ^2^) between adiposity (%fat, measured by dual‐energy X‐ray absorptiometry) and increments in shape index in the form of *W/H* ^*β*^. The sample is the same as shown in Figure 1.^25^

• Cole's first approach is to create a series of indices, $W/H^{\beta }$, by varying values for β (e.g., β = 1.0, 1.2, etc.) while examining correlations of the derived indices with height and adiposity; the ideal index would be uncorrelated with height and maximally correlated with percent fat. An example of this approach is shown in Figure 5 for the adult male NHANES participants presented earlier. The *R* ^2^ values for height versus shape index ( $W/H^{\beta }$) form a curvilinear line that approaches zero when the value for β is slightly larger than 2.0 (panel A). The vertex of the curve can be found by fitting the data to a parabolic curve (i.e., modeling correlation = *β* + *β* ^2^), finding the first derivative of the model, setting the derivative equal to 0, and solving for β.^66^ This vertex occurs at 2.17. When percent fat, measured with reliable and validated dual‐energy X‐ray absorptiometry, is plotted as a function of weight–stature index power (panel B), the vertex found using the method described above occurs at a β slightly less than 2.0, 1.93. At this value of β, the maximum *R* ^2^ was 0.714. The maximum *R* ^2^ in this example for percent fat versus $\frac{W}{H^{\beta }}\left(0.714\right)$ is similar in magnitude to the *R* ^2^ values reported by Keys in their study (0.521–0.790),^2^ thus leaving about one‐third to one‐half of between‐individual differences in adiposity unaccounted for by the optimum weight–stature index.

- Cole's second suggested method^5^ is to solve Equation (4), $\log_{10}W=\log_{10}\alpha +\beta \log_{10}H+\log_{10}\varepsilon$, for β as shown earlier for the adult male NHANES sample depicted in Figure 1. The regression line slope (i.e., β ± SE) in this example is 2.09 ± 0.016. The exponent of *β*, 2.09 found using Cole's second method, is very close to the exponent 2.0, which, as shown above, provides a weight–stature index *W/H* ^*β*^ independent of height and maximally correlated with adiposity.
- Cole's third approach, originally suggested by Benn,^3^ gives a value for β ± SE in the weight–stature index $W/H^{\beta }\text{minimal}$ly correlated with height and maximally correlated with adiposity. There are two steps, first calculation of the slope (*m*) of the regression of weight on height in the sample (note that this regression is conducted on the original data not the log_10_ transformed data) and second, finding the average height (*H*_0_) and weight (*W*_0_) of the sample. Then

(5) $$\beta =m\times \left(H_{0}/W_{0}\right)$$

The standard error of *β* can be calculated with an equation suggested by Benn.^3^ The value for *β* derived in the adult male NHANES sample shown in Figure 1 using Benn's method is 2.17. Benn would likely have rounded this value to 2 when applied in actual studies as a means of “making computation easy.”^3^ The sequence of steps showing why Benn's equation for deriving a value for *β* makes the index *W/H* ^*β*^ largely independent of height is presented in Supporting Information IV.

**FIGURE 6:**
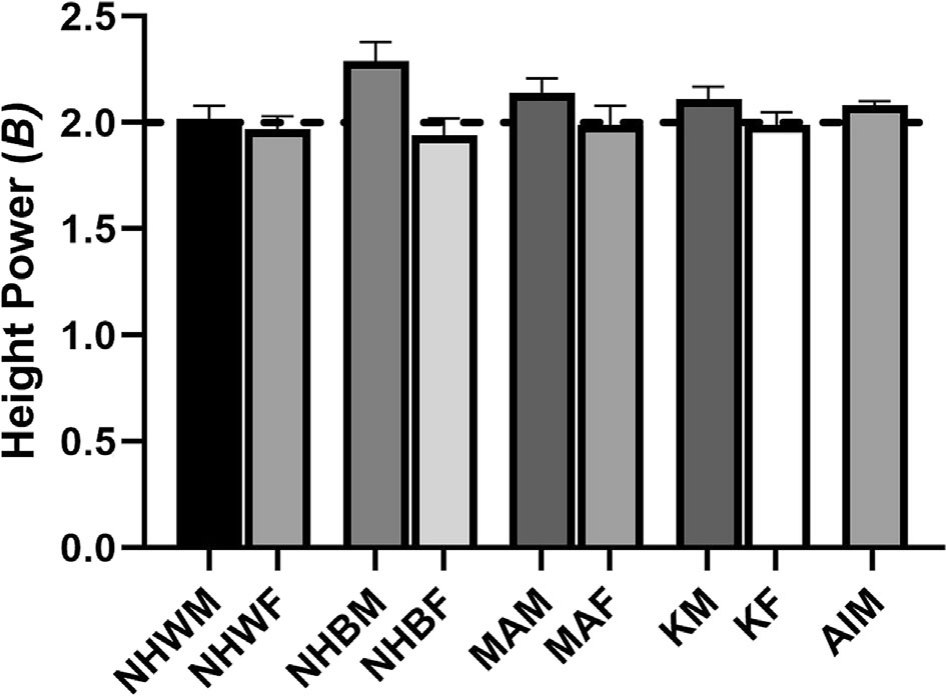
Powers (*β* ± SE) of weight scaled using the allometric power rule, 𝑦 = α𝑋^𝛽^ε. The figure contains data for non‐Hispanic white and black males (NHWM, NHBM) and females (NHWF, NHBF), Mexican American males and females (MAM, MAF), Korean males and females (KM, KF), and Asian Indian males (AIM). The powers plotted were obtained by regressing, for each group, the log_10_ weight on log_10_ height, 𝑙𝑜𝑔_10_ (𝑤𝑒𝑖𝑔ℎ𝑡) = 𝑙𝑜𝑔_10_ (ℎ𝑒𝑖𝑔ℎ𝑡), resulting in an equation 𝑙𝑜𝑔_10_ (𝑤𝑒𝑖𝑔ℎ𝑡) = 𝑙𝑜𝑔_10_ (𝛽_0_) + 𝛽_1_𝑙𝑜𝑔_10_ (ℎ𝑒𝑖𝑔ℎ𝑡). The values plotted are the values of *β*_1_ so obtained for each group. Within‐sex group differences in values for *β* were nonsignificant in the NHANES studies; the values for *β* differed significantly from 2.0 in NH black and Mexican American males. Data are from the US and Korean NHANES^25^ and from the anthropological survey of India.^67^

While only the first method, described above, clearly demonstrated satisfaction of the two criteria, a metric independence of height and maximum correlation with adiposity, both the first and second methods give values for β that are very close to 2.0 in this sample, as in BMI. However, any difference between the actual value for β and 2.0 implies that BMI in the sample will not be independent of height. Although the value of β in our example was very close to 2.0 (i.e., 2.09), the small difference leads to a positive nonsignificant slope when BMI is plotted against height for which β = 2.0; that is, BMI will scale to height as *H* ^*β* in the evaluated sample − 2.0^ or as H^0.09^. Values for β at or very close to 2.0 are published for adults in many previous studies as, for example, in the NHANES and Korean NHANES males and females, and Asian Indian males shown in Figure 6 reported earlier^25^, ^67^ and the Gerontology Research Table created by Andres ( β 2.0 for men and women). Adult females, in these examples and in other reports,^8^, ^25^ almost always have values for β that are slightly smaller (e.g., Figure 6, 1.94–1.99) than males (2.02–2.29). The value of β in the NH Black males (2.29) was nonsignificantly larger than their NH White (2.02), Mexican American (2.14), and Korean (1.99) NHANES male counterparts. The value of β is thus often not exactly 2.0 in adult samples, an effect that will contribute to variable magnitude weight classification bias when grouping BMI categories using single‐value thresholds. The magnitude of bias will be small when the between group differences in the best height exponents are small such as those seen in this figure. In this case, rounding values of β to 2 when deviations in measured powers are small can be justified for convenience of calculation and application. Adiposity also varies for the same BMI across the adult age span within the same sample,^68^ between race/ethnic groups at the same sex and age,^25^, ^69^ and between individuals of the same sex, age, and race/ethnicity who differ in muscularity.^70^ Further studies in populations underrepresented in studies to date to assess the magnitude of the bias are needed. Body mass index is but one tool for assessing relative weight for height. Other metrics such as partitioning of tissue types (e.g., fat‐mass vs. fat‐free mass), location of fat deposition (e.g., intra‐abdominal vs. extra‐abdominal or central vs. peripheral), fitness (e.g., VO_2_ max, heart rate recovery), and plasma glucose and lipid concentrations, all of which are now relatively easily available, should be utilized to better understand the implications of a given person's BMI.

The mathematical paradigm presented in our review is grounded on the observation that percent fat is independent of height in adults. That is not the case in children and adolescents, a topic reviewed in detail by Peterson et al.^71^ For example, youths with obesity are taller than their nonobese peers early in adolescence, an observation giving rise to significant correlations between percent fat and height. Accordingly, the optimum value of β for predicting percent fat with a weight–stature index under these circumstances can be derived as

(6) $$\text{Percent}\;\mathrm{fat}=W/H^{\beta }$$

with β on a specific sample calculated as

(7) $$\beta =\log\;\left(W/\text{percent}\;\mathrm{fat}\right)/\log\;\left(H\right)$$

Values of *β* evaluated in pediatric population samples using Equation (6) range from about 2 to 3,^71^ an observation made by Quetelet in the 1842 translation^1^ of his 1835 publication.^17^

## 4 RELATED INDICES

Many other allometric relations in humans are reported in the medical literature, and some form the basis of widely used indices for which the same mathematical and development concepts apply as in the current report. Some published indices of this type include total body fat mass/*H* (males) and *H* ^0.8^ (females)^72^; skeletal muscle, fat‐free mass, and appendicular lean mass/*H* ^2^ ^73^, ^74^, ^75^; brain mass/*H* ^0.5^ ^76^; left ventricular mass/*H* ^1.7^ ^77^; waist circumference/*H* ^0.5^ ^78^, ^79^, ^80^; and sagittal diameter/*H*.^81^ Although these indices are all formulated around the same allometric concepts, each has unique development pathways and mathematical nuances.

## 5 CONCLUSIONS

Body mass index, $W/H^{2}$, has a firm mathematical foundation, is elegantly simple and easy to calculate, is independent of height, and, relative to other power‐type weight–stature indices, is maximally correlated with measures of adiposity in adults. These properties of BMI make it useful as one component in the evaluation and monitoring of people with overweight and obesity in research and clinical settings. Despite these laudable properties, body weight and height together can only account for one‐half to two‐thirds of between‐individual differences in adiposity, an observation prompting the suggestion to include additional measures in some patient evaluations that improve percent fat and health‐risk predictions.^11^, ^70^, ^80^, ^82^

## AUTHOR CONTRIBUTIONS

Authors' contributions to manuscript: SBH, JDS, DMT, SY, MH, CM, JB, and AP designed review outline; SBH provided essential materials; SBH, JDS, DMT, SY, and MH analyzed data; SBH, JDS, DMT, SY, MH, CM, JB, and AP wrote the paper; and all authors had primary responsibility for final content.

## CONFLICT OF INTEREST STATEMENT

SBH serves on the Medical Advisory Boards of Tanita Corporation, Novo Nordisk, Abbott, Regeneron, and Medifast. The authors and their close relatives and their professional associates have no financial interests in the study outcome, nor do they serve as an officer, director, member, owner, trustee, or employee of an organization with a financial interest in the outcome or as an expert witness, advisor, consultant, or public advocate on behalf of an organization with a financial interest in the study outcome.

## NAMES FOR PUBMED INDEXING

Brown, Heo, Heymsfield, McCarthy, Pietrobelli, Thomas, Yang.

## Supporting information


**Data S1.** Scaling Relations and Body Proportions.
Data S2. Gould's Analysis of How Weight Scaled to Height in Soldiers.
Data S3. Power law models for the 1959 Metropolitan Life Insurance Company desirable weights.
Data S4. Sequence of steps showing why Benn's equation for deriving a value for β makes the index W/H^β^ largely independent of height.
