# Supplementary material for: Weight/height2: Mathematical overview of the world's most widely used adiposity index
Source: Obes Rev. 2024 Oct 10;26(1):e13842. doi: 10.1111/obr.13842 (PMC11611441; doi:10.1111/obr.13842)
Supplement: Supplementary file 1 — Data S1. Scaling Relations and Body Proportions. Data S2. Gould's Analysis of How Weight Scaled to Height in Soldiers. Data S3. Power law models for the 1959 Metropolitan Life Insurance Company desirable weights. Data S4. Sequence of steps showing why Benn's equation for deriving a value for β makes the index W/Hβ largely independent of height. [file OBR-26-e13842-s001.pdf]

SUPPORTING INFORMATION

Weight/Height^2: Mathematical Overview of the World's Most Widely Used Adiposity Index

Steven B. Heymsfield[1], John D. Sorkin[2,3], Diana M. Thomas[4], Shengping Yang[1], Moonseong Heo[5], Cassidy McCarthy[1], Jasmine Brown[1], Angelo Pietrobelli[6]

[1]Pennington Biomedical Research Center, LSU System, Baton Rouge, LA, USA; [2]Baltimore VA Medical Center Geriatric Research, Education and Clinical Center, USA; [3]University of Maryland School of Medicine, Department of Medicine, Division of Gerontology, Geriatrics, and Palliative Care, USA [4]Department of Mathematical Sciences, United States Military Academy, West Point, NY, USA; [5]Department of Public Health Sciences, Clemson University, Clemson, South Carolina, USA; [6]Verona University Medical School, Verona, Italy

Address Correspondence to:

Steven B. Heymsfield, M.D.
Pennington Biomedical Research Center
6400 Perkins Road
Baton Rouge, LA 70808
Tel: 225-763-2541
Fax: 225-763-3030
E-mail: Steven.Heymsfield@pbrc.edu

Supporting Information I. Scaling Relations and Body Proportions.
Supporting Information II. Gould's Analysis of How Weight Scaled to Height in Soldiers.
Supporting Information III. Power law models for the 1959 Metropolitan Life Insurance Company desirable weights.
Supporting Information IV. Sequence of steps showing why Benn's equation for deriving a value for β makes the index W/H$^\beta$ largely independent of height.

Supporting Information I. Scaling Relations and Body Proportions.

Not all body regions increase proportionally with greater height. These proportional differences are reflected in the power of regional weights (W) when scaled to height (H). For example, if regional weight 1 (W1) scales to height with a power of β1,

$$W1 = \alpha_1 (H)^{\beta 1} \qquad (1)$$

and total body weight (W2) scales to height as

$$W2 = \alpha_2 (H)^{\beta 2}, \qquad (2)$$

then

$$W1/W2 = \alpha_1/\alpha_2 (H)^{\beta 1 - \beta 2} \qquad (3)$$

Equation 3 shows how the proportion of body weight (W2) as W1 changes with height (i.e., β1-β2). When β1 and β2 are equal (i.e., when both weights scale the same to height), the value of β1−β2 is 0 and a non-zero number raised to the power of zero equals 1. When the difference between β1 and β2 is at or near zero no correlation will be observed between the ratio of the two weights and height. If the difference observed between β1 and β2 is not zero, the ratio of W1 to W2 will scale positively or negatively to height.

Supporting Information II. Gould's Analysis of How Weight Scaled to Height in Soldiers[†].

Gould organized his samples of White men according to height in ½ inch increments as shown in **Table IX**. He derived the weight/height ratio, and as shown in the table W/H increased with greater height. That led Gould to examine W/H^2. He first derived the "modulus" (mean) of the whole sample for weight/height^2 as 0.03156. Gould then reasoned that if the mean weight/height^2 (analogous to BMI) was independent of height, he could compute the weight in men at each height as 0.03156 x group height^2. Gould then compared his "hypothetical" weight to the observed weight shown in **Table IX** and those results are shown in **Table X**. There was no systematic difference between computed and observed weights in relation to height, leading Gould to surmise that weight in these soldiers increased in proportion to height^2. Reproduced with permission from the publisher.

TABLE IX.

Aggregate Mean Weight of White Men, by Height, and Ratio to Stature.

Height	Number of Men	Weight	Pounds to Inch	Height	Number of Men	Weight	Pounds to Inch
in.		lbs.		in.		lbs.	
60	24	111.79	1.863	68	1 246	145.71	2.143
60½	44	115.78	1.914	68½	1 248	148.32	2.165
61	57	119.00	1.951	69	917	150.02	2.174
61½	86	119.68	1.946	69½	836	152.23	2.190
62	172	121.31	1.957	70	633	154.46	2.207
62½	217	120.98	1.936	70½	497	157.24	2.230
63	294	124.40	1.975	71	348	159.60	2.248
63½	455	126.59	1.994	71½	269	162.11	2.267
64	576	129.88	2.029	72	200	162.97	2.263
64½	816	131.43	2.038	72½	140	165.04	2.276
65	820	132.81	2.043	73	92	168.46	2.308
65½	1 172	135.14	2.063	73½	56	168.11	2.287
66	976	137.16	2.078	74	45	170.08	2.298
66½	1 358	139.32	2.095	74½	17	173.47	2.328
67	1 283	142.31	2.124	75	14	166.66	2.222
67½	1 483	144.27	2.137	Over 75	23	173.75	2.286

TABLE X.

Theoretical Weight for different Statures, and Comparison with Observation.

Height	Computed Weight	Difference Comp. — Obs.	Height	Computed Weight	Difference Comp. — Obs.
in.	lbs.	lbs.	in.	lbs.	lbs.
60	113.62	+ 1.83	68	145.94	+ 0.28
60½	115.52	− 0.26	68½	148.09	− 0.23
61	117.44	− 1.56	69	150.26	+ 0.24
61½	119.37	− 0.31	69½	152.45	+ 0.22
62	121.32	+ 0.01	70	154.65	+ 0.19
62½	123.28	+ 2.30	70½	156.87	− 0.37
63	125.27	+ 0.87	71	159.10	− 0.50
63½	127.26	+ 0.67	71½	161.35	− 0.76
64	129.27	+ 0.61	72	163.61	+ 0.64
64½	131.30	− 0.18	72½	165.89	+ 0.85
65	133.34	+ 0.53	73	163.19	− 5.27
65½	135.40	+ 0.26	73½	170.50	+ 2.39
66	137.48	+ 0.32	74	172.83	+ 2.75
66½	139.57	+ 0.25	74½	175.17	+ 1.70
67	141.68	− 0.63	75	177.53	–
67½	143.80	− 0.47	Over 75	–	–

†**Table IX** is from page 408 and **Table X** is from page 409 of Gould's report (1).

(1) Gould BA. Investigations in the military and anthropological statistics of American soldiers. Cambridge: Riverside Press 1869.

Supporting Information III. Power law models for the 1959 Metropolitan Life Insurance Company desirable weights for men and women.

The Metropolitan Life Insurance Desirable Weight tables, published in 1959, were based on the 1959 Build and Blood Pressure Study conducted by the Society of Actuaries (1, 2). The Build Study reported summary statistics, means, of the heights (by increments of one inch), and weights (in pounds) and vital status of policy holders of several million life insurance policies written by twenty-six large life insurance companies, 1935-1953, in the US and Canada (2). (The unit of observation was a policy, not a person. A given person might have more than one policy and so the person would appear more than once in the Build Study.) Mortality follow-up continued until 1954 (3, 4).

The Metropolitan desirable weight tables, one for men and a second for women, give a range of desirable weights, by inches of height (women from 4'10" to 6'0"; men 5'2" to 6'4") for each of three frame size categories for men and women 25 to 59 years of age. No guidance was given on how to determine frame size. Measurements are given assuming indoor clothing and shoes (3). We converted the heights and range of weights (in pounds) in the Metropolitan Tables to meters and kilograms. Within sex, for each frame size, we calculated, the mean of the weight range, W_{Mean} , for each height, H. Assuming a power function related weight to height (i.e., $W_{Mean} = \alpha H^{\beta}$) we $\log_{10}$ transformed the equation, $log_{10}(Weight_{Mean}) = log_{10}(\alpha) + \beta log_{10}(Height)$, regressed $log_{10}(Weight_{Mean})$ on $log_{10}(Height)$ and obtained a value for β, the exponent of height and $log_{10}(\alpha)$, the intercept. A total of six separate regressions were run for each of the three frame sizes for men and women. In the table below we report β, the Scaling Exponent (Power) and the $10^{log_{10}(\alpha)}$ the proportionality constant of the power function for each of the three frame sizes for men and women.

β, scaling exponents (power) of Height and α, Proportionality Constants, of a Power Function Relating Height to the Mean of the Desirable Weight Range Presented in the Metropolitan 1959 Desirable Weight Tables.

**Model** (Frame Size)	**Men**	**Women**
**Small**	$W = 0.0033 \times H^{1.91}$	$y = 0.0031x^{1.91}$
**Medium**	$W = 0.0039 \times H^{1.89}$	$y = 0.0036x^{1.89}$
**Large**	$W = 0.0053 \times H^{1.84}$	$y = 0.0067x^{1.79}$

The table, below, summarizes the low and high body mass indices (BMI) according to sex and frame size.

Range of Desirable BMIs by Sex and Frame Size Computed Using Heights and Mid-Point of Weight Ranges in the 1959 Metropolitan Live Insurance Desirable Weight Tables

	**Men**		**Women**	
	Low	High	Low	High
**Frame**	kg/m^2			
Small	20.7	21.3	19.4	19.9
Medium	22.1	22.6	20.6	21.3
Large	23.5	24.5	22.3	23.4

(1) Metropolitan Life Insurance Company. New weight standards for men and women. Stat Bull Metrop Insur Co. 1959;40:1–4.
(2) https://archive.org/details/sim_statistical-bulletin-sb_november-december-1959_40
(3) Chichester S, Holmes TM, Hubbard J. Ideal body weight: A commentary. Clin Nutr ESPEN. 2021;46:246-250. doi:10.1016/j.clnesp.2021.09.746.
(4) Komaroff M. For Researchers on Obesity: Historical Review of Extra Body Weight Definitions. J Obes. 2016;2016:2460285. doi:10.1155/2016/2460285.

Supporting Information IV. Sequence of steps showing why Benn's equation for deriving a value for β makes the index W/H^{β} largely independent of height.

The concept of developing a relative weight index, as articulated by Benn (1), is based on the assumption that a linear relationship exists between a "standard" body weight (W_S) and height (H). A linear relationship also holds for the corresponding logarithmic values, consistent with the Taylor series approximation.

Based on this concept, the standard form relating Ws to H can be written as,

$$W_s(H) = W_0 + m(H - H_0), \quad (1)$$

where W_0 and H_0 are central measures (e.g., mean) of standard weight and height in a sample, and m is the slope of the regression of weight against height in the sample. We can then take the logarithm of both sides,

$$\log[W_s(H)] = \log[W_0 + m(H - H_0)], \quad (2)$$

and by Taylor expansion,

$$\approx \log(W_0) + \frac{m}{W_0}(H - H_0) \quad (3)$$

Since we plan to later exponentiate the function (equation 7) to transform it back to the original scale, we can approximate H and H_0 using $\log(H)$ and $\log(H_0)$. The goal is to simplify the exponential term, allowing the logarithm and exponential functions to cancel each other out.

Similarly, by Taylor expansion at H_0,

$$\log(H) \approx \log(H_0) + \frac{H - H_0}{H_0} \quad (4)$$

and thus,

$$H_0[\log(H) - \log(H_0)] \approx H - H_0 \quad (5)$$

Substituting into equation 3,

$$\log[W_s(H)] \approx \log(W_0) + \frac{m}{W_0} H_0[\log(H) - \log(H_0)] \quad (6)$$

Now, exponentiate both sides,

$$W_s(H) \approx W_0 \left[e^{\log(H)-\log(H_0)}\right]^{\frac{m}{W_0}H_0} \quad (7)$$

$$W_s(H) \approx W_0 \left(\frac{H}{H_0}\right)^{\frac{m}{W_0}H_0} \quad (8)$$

We let $B = \frac{m}{W_0} H_0$, then,

$$W_s(H) \approx W_0 \left(\frac{H}{H_0}\right)^B = W_0 \frac{H^B}{H_0^B} \quad (9)$$

For a specific weight, we can standardize it by $W_s(H)$, that is,

$$\frac{W}{W_s(H)} \approx \frac{W}{W_0 \frac{H^B}{H_0^B}} = \frac{W H_0^B}{H^B W_0} = \frac{W}{H^B} \frac{H_0^B}{W_0} \quad (10)$$

Given a population/sample, $\frac{H_0^B}{W_0}$ is a constant, and thus the index, $\frac{W}{H^B}$, is approximately proportional to body weight divided by the standard weight for height, effectively normalizing for height. Note that the same applies if a base-10 logarithm is used.

1. Benn RT. Some mathematical properties of weight-for-height indices used as measures of adiposity. *Br J Prev Soc Med*. 1971; 25: 42-50.
